# Milk-Sickness

**Published:** 1858-05

**Authors:** J. P. De Bruler

**Affiliations:** Rockport, Ind.


ARTICLE III.

### MILK-SICKNESS.

BY J. P. DE BRULER, M.D., ROCKPORT, IND.

DR. BYFORD,

SIR,—I propose briefly to comply with your request with reference to milk-sick. Dr. Crooks informs me that he has written to you upon the subject, and as his views on the pathology and treatment of the disease mainly correspond with my own, I will confine my remarks principally to its *etiology.* I assume that the disease is produced by an *animal* poison, and offer the following reasons in support of that position.

In the first place, it would probably be profitable to see what it is not. It is certainly not any mineral or vegetable poison, with the properties of which we are acquainted. Poisons of these two classes usually produce death by their caustic or excoriating effects, as arsenic, by their narcotic, as opium, or by a violent and overwhelming effect upon the nervous system, as strychnine, etc. But in no instance do we know of their possessing the power of accumulation *per se.* They have not the vital principle of development. There can never at any time be more in the system than was taken in by the animal. Hence, an animal which should receive enough of this poison into her system as to produce death, could not by her flesh or secretions destroy a dozen equally as strong and healthy as she was; much less could we suppose, that the animals which received the secondary poison could be capable of producing the same destructive results.

Now, if the poison which produces milk-sickness does possess this accumulative power, are we not driven to the conclusion that it *is not* any vegetable or mineral poison with which we are acquainted. But can we sustain the hypothesis that it is an animal poison? I do not pretend to say that we can, but analogy now comes to our aid, and at least favors the supposition. Some animal poisons do not possess this vital principle of reproduction, as for instance, those poisons generally called venoms. The poison of the viper, hornet, etc., are undoubtedly the result of organization; but its vitality is of so low a grade (if you will allow the expression), that they do not possess the power of reproduction.

But there is a large class of animal poisons commonly called infections, which do possess the power of self-propagation, whenever they are placed in a proper condition for such development. One rabid dog could probably infect a score or more, and they in turn produce the disease in an equal ratio.

The same is true of small-pox, measles, psora, etc. Now, itch is known to be the result of an insect which is capable of reproduction to an indefinite extent. We do not know that all infectious diseases are thus propagated, but there is something in their manner of expansion which would seem to favor the opinion that they are. In the first place, we must come in contact with them in some way, either by direct contact, or through the atmosphere. And then there is a period of incubation, which has a greater or less relation to a fixed time. For instance, nobody ever took small-pox in five days, nor probably in two or three weeks after exposure to the infection. A certain time seems necessary for the development of the disease, or what, to my mind, is more probable, for infusorial development. If the disease is not infusorial, how can we account for the fact, that it is so much more dangerous when received by inhalation than by inoculation? If the disease is produced by animalcules, we may, with some plausibility, account for this difference by the hypothesis, that these animalcules are essentially different, or more probably, in different stages of their development, from those floating in the atmosphere, possessing some peculiar power capable of producing the more violent result. Dr. Mussey saw and described very carefully infusorial in a drop of condensed vapor near a cholera patient, and these seemed to be in various stages of development.

Now you can elaborate this idea without my saying more about it, as well as I can. Infectious diseases are sometimes the result of animalcule. May they not *always* be produced in the same way? Is not the fact, that these poisons possess the power of self-accumulation, proof of this position? Now, have we any proof that the poison of milk-sick bears any relation to any of these animal poisons? It seems to me that the principle which bears a fixed and acknowledged relation to all infectious diseases, should be our starting point. Is milk-sick, or the poison of milk-sickness, like the poison of all infectious diseases, accumulative? If so, is not this in obedience to a vital law? And is not the *infusorial* hypothesis more reasonable than that our systems do somehow or other possess the power of manufacturing this poison?

With reference to the accumulative power of the milk-sick VIRUS, I will relate the following fact which fell under my own observation:—A gentleman of this town had a cow which run at large, caught the disease, and gave it to her calf. The calf died, and his dogs eat the flesh of the calf; they, in a few days, sickened, with all the ordinary symptoms of TIRES, and both died. A pet crow was seen eating the dogs, in a few days it refused to eat, could scarcely be made to hop or fly, as was its custom when in health, and in a day or two died. I should have been glad to have pursued this subject further, but the crow was thrown away by some of the family before I knew that it was dead. It was, however, conclusive proof to my mind that the poison had passed through four successive animals (if you call a crow an animal) and lost none of its virulence.

Do not the above facts to some extent refute the theory of its production by the rhus toxicodendron, arsenic, or any known vegetable or mineral poison with which we are acquainted. True, the rhus toxicodendron, and probably many other plants, may afford the proper nisus for these infusory animals or insects, and may supply them with appropriate food for their development, and in this way many diseases that are now acknowledged to be infectious may have had their origin.

Of the pathology of the disease I do not profess to know anything. I have never made a *post-mortem* examination. The late Dr. S. P. Cisna, of this place, informed me that he had twice. One was a case that died in a few days after the attack. In this he found the usual evidences of an active inflammation of the stomach, and particularly of the pyloric region. The other was a protracted case (I forget how long). In this he found the pyloric orifice and most of the duodenum so thickened and contracted that he could scarcely pass a probe through this outlet of the stomach.

I need say nothing in relation to the treatment, for I have heretofore given you my views and experience; and I may have given you the opinions above indicated, but if so, I have forgotten it.

